# Incidence of Gastrointestinal Neuroendocrine Tumor: Case Series, Armed Forces Hospital Southern Region, Hospital-Based Tumor Board Registry

**DOI:** 10.1155/2020/8819392

**Published:** 2020-10-20

**Authors:** Abdullah Mohammed Albishi, Ahmed Mostafa Mohamed Mostafa, Hatim Mahgoub Ali, Yahia Atiah Alhagawi, Mohamed F. Bazeed, Mahmoud R. A. Hussein, Elshfeia Elhag Mohmed Ali Aloba, Ahmed Youssef Aboelyazid

**Affiliations:** ^1^Gastroenterology and Endoscopy Department, Armed Forces Hospital Southern Region, Khamis Mushayt, Saudi Arabia; ^2^Clinical Oncology, Faculty of Medicine, Ain-Shams University, Egypt; ^3^Oncology Department, Armed Forces Hospital Southern Region, Khamis Mushayt, Saudi Arabia; ^4^Radiology Department, Armed Forces Hospital Southern Region, Khamis Mushayt, Saudi Arabia; ^5^Pathology Department, Armed Forces Hospital Southern Region, Khamis Mushayt, Saudi Arabia; ^6^Preventive Medicine Department, Armed Forces Hospital Southern Region, Khamis Mushayt, Saudi Arabia

## Abstract

Neuroendocrine tumors are aggressive and rare tumors which can occur almost everywhere in the body. The annual incidence of neuroendocrine tumors is 2.5-5 per 100000. We report seven cases of gastrointestinal neuroendocrine tumors which were diagnosed and treated at our hospital from the time period of 2016-2018 knowing that the total number of our hospital tumor board cases registry during the same period was 444 cases.

## 1. Introduction

Generally, Neuroendocrine tumors (NETs) are rare tumors comprising ~2% of all malignancies [1] with the gastrointestinal tract and the lung as the most common sites [2].

Neuroendocrine tumors (NETs) are a heterogeneous group of epithelial neoplastic proliferations arising in many body organs. Irrespective of their primary site and of their grade of differentiation, neoplastic cells share features of neural and endocrine differentiation: the “neuro” property is based on the identification of dense core granules that are similar to dense core granules (DCGs) present in serotonergic neurons, which store monoamines, and the “endocrine” property refers to the synthesis and secretion of these monoamines which is about two-thirds of NETs that arise in the gastrointestinal tract and pancreas [1, 3].

## 2. Case Reports

This case series describes seven cases of gastrointestinal NETs diagnosed and presented in the tumor board at our hospital from the time period starting from 01/01/2016 till 31/12/2018 as shown in Table 1. Out of seven cases, four were male and three were females. Four patients out of seven present beyond 60 years of age. Appendix is the most common site of NETs in our hospital.

### 2.1. 1st Case

A 60-year-old male presented with abdominal pain and vomiting with constipation for 4 days; CT of the abdomen showed an enhancing mass at the ileocecal area including the appendix; he was diagnosed as having complete intestinal obstruction and underwent right hemicolectomy; histopathology report showed goblet cell carcinoid of the appendix. The patient is doing well with follow-up with an oncologist.

### 2.2. 2nd Case

A 20-year-old female presented with right iliac fossa abdominal pain for 2 days, was diagnosed as having acute appendicitis, and underwent appendectomy; histopathology report showed a well-differentiated neuroendocrine tumor of the appendix. Patient is doing well with follow-up with an oncologist.

### 2.3. 3rd Case

A19-year-old female presented with right iliac fossa abdominal pain for 1 day, was diagnosed as having acute appendicitis, and underwent appendectomy; histopathology report showed a well-differentiated neuroendocrine tumor of the appendix grade 1. Patient is doing well with follow-up with an oncologist.

### 2.4. 4th Case

**Figure 1:**
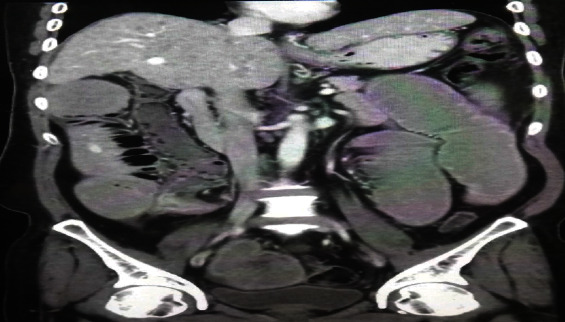
Case 4. Coronal postcontrast CT scan study showed a collapsed cecum with dilated ileum and enhanced lesion at the ileocecal valve.

A 52-year-old male presented with abdominal pain and vomiting with constipation for 3 days; CT of the abdomen showed an enhancing mass at the ileocecal area including the appendix as shown in Figure 1; she was diagnosed as having complete intestinal obstruction and underwent right hemicolectomy; histopathology report showed a well-differentiated neuroendocrine tumor of the small bowel. Patient is doing well with follow-up with an oncologist. Follow-up CT of the abdomen showed a free anastomosis site, with follow-up with an oncologist.

### 2.5. 5th Case

**Figure 2:**
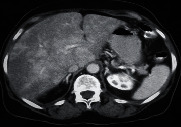
Case 5. Axial postcontrast CT scan showed patchy heterogeneous enhancement of liver metastasis.

An 80-year-old female presented with abdominal pain and weight loss for 4 months; ultrasound of the abdomen showed multiple liver lesions. CT of the abdomen showed multiple liver lesions with an enlarged appendix as shown in Figure 2; she underwent liver biopsy, and histopathology report showed a metastasis of the neuroendocrine tumor. The patient died.

### 2.6. 6th Case

A 22-year-old male presented with right iliac fossa abdominal pain for 2 days, was diagnosed as having acute appendicitis, and underwent appendectomy; histopathology report showed a low-grade neuroendocrine tumor of the appendix. Patient is doing well with follow-up with an oncologist and planned for right hemicolectomy.

### 2.7. 7th Case

An 86-year-old male presented with vomiting of blood for 2 days, underwent gastroscopy, and showed ugly-looking gastric ulcer; histopathology report showed a low-grade neuroendocrine tumor. Patient is following up with an oncologist.

## 3. Results and Discussion

As neuroendocrine cells are distributed among the whole body, so NETs have been described in multiple organs such as the central nervous system, respiratory tract, the larynx, gastrointestinal (GI) tract, thyroid, skin, breast, and urogenital system. The GIT and lung are the most common primary sites for NET [1, 2]. This type of neoplasia has substantial variations in both tumor biology and clinical presentation; the biology of each NET depends on its primary tumor localization, cellular morphology, and mitotic activity, and clinically, NET may manifest by the expression of autonomous hormone secretion of either a peptide hormone or biogenic amine [4].

The pathologic classifications of neuroendocrine tumors across different organ systems use a range of site-specific terminologies and criteria, creating significant confusion among pathologists and treating clinicians [5]. The most widely used is the WHO system for classification of NET which is frequently updated, and at the time of starting our case series, we were using the WHO version 4 for classification of neuroendocrine tumors [6] and according to this classification, NETs are classified into three grading subgroups based on the mitotic activity and Ki67 immunostaining: G1 (mitotic count < 2/10 HPF and/or Ki − 67 index < 3%), G2 (mitotic count 2-20/10 HPF and/or Ki-67 index 3-20%), and G3 (mitotic count > 20/10 HPF and/or Ki − 67 index > 20%). G1 and G2 NETs have well-differentiated morphology and are referred to as G1 or G2 neuroendocrine tumors (NET), while G3 NETs are considered poorly differentiated and referred to as neuroendocrine carcinomas (NEC) [4]; one of the main advantages of this system is the higher ability to determine prognosis [7]. The total number of NETs diagnosed and presented in the tumor board at our hospital from the time period starting from 01/01/2016 till 31/12/2018 was 11 out of 444 total cases representing almost 0.03%, and this is of low incidence if compared to the usual global incidence [8], whereas the total number of GIT NETs was 7 cases, representing almost 65% of the all NET cases diagnosed at our hospital. Most of our cases were appendicular NETs (6 out of the 7 cases), and all these cases were of low grade (grade 1). There was slight male predominance representing 4 out of 7 cases, and 3 out of 7 patients were beyond 60 years of age.

We have to mention that the term carcinoid has been criticized because it was used to describe different tumors distinct in their etiology, prognosis, and management, leading to terminological confusion and diagnostic unreliability [1], and also, this term is considered to be a straight misnomer as the malignancy of this tumor group can be confirmed on the basis of local invasion prior to metastases [9] and not benignly as Oberndorfer mistakenly assumed [1], and so the term carcinoid should be used whenever there are the symptoms of this syndrome which are watery diarrhea, flushing, bronchospasm, hypotension, and right-sided heart disease that correlates with serotonin hypersecretion since properties of serotonin include vasodilation, bronchoconstriction, and smooth muscle contraction [10]. In our case series, no single case presented with secretory symptoms and all of them presented with either a clinical picture of acute appendicitis or right hypochondrial pain.

### 3.1. Diagnosis

**Figure 3:**
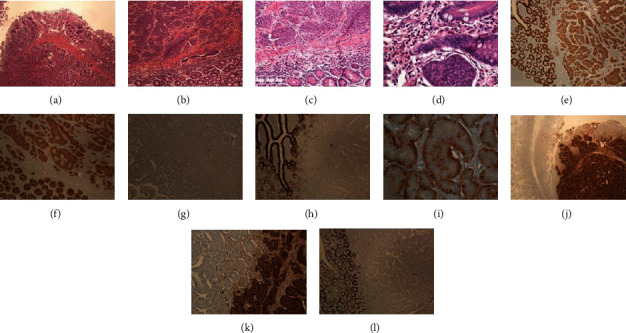
Histopathology features of one of the cases, well-differentiated neuroendocrine tumor of the small intestine (ileum), classic type: (a–c) the histological sections show tumor cells with minimal pleomorphism arranged in cording and nesting growth patterns, extensively involving the mucosa, submucosa, and muscle layer. The main bulk of the tumor is seen in the submucosa and the muscle layer. The tumor cells have stippled chromatin, inconspicuous nucleoli, and slightly granular eosinophilic cytoplasm. There is no significant mitotic activity, and the tumor cells show little pleomorphism. There is no cell necrosis (H&E stains: (a) ×4, (b) ×10, (c) ×20, and (d) ×40). The tumor cells were positive for the epithelial markers CDX-2 ((e) ×10) and pancytokeratin ((f) ×10) and negative for CK7 ((g) ×10) and CK20 ((h) ×10). The tumor cells were diffusely and strongly reactive for the markers of neuroendocrine differentiation including both synaptophysin ((i) ×40) and chromogranin ((j) ×4 and (k) ×10). Ki67 labelling index was very low (less than 1%, (l) ×10).

The improvement of the current diagnostic techniques has led to an increased number of patients diagnosed with GIT NET, and, in contrast with the past, most of the tumors belong to the so called “nonfunctioning” category and not associated with symptoms and signs of hormone hypersecretion [3]. The diagnosis of NET includes different modalities, but proper tumor localization is essential because surgery is still the cornerstone modality of treatment of nonmetastatic NET [11]. Several imaging methods are available including CT, magnetic resonance imaging, ultrasonography, scintigraphy, and positron emission tomography [1]; following imaging and localization, biopsy should be obtained for histopathological diagnosis as shown in Figure 3 and this may include performing upper endoscopy and colonoscopy with ileoscopy for gastrointestinal NET [7].

Commonly measured tumor markers in NETs include serum CgA and 5-HIAA, the final secreted product of serotonin, levels in a 24-hour urine sample [12], and actually, serum CgA is more sensitive and broadly applicable marker than urinary 5-HIAA because it does not depend on serotonin secretion so it is preferred over 5-HIAA for bronchial and rectal tumors, because they do not generally secrete serotonin [13]; also, plasma CgA levels correlate with tumor bulk, differentiation, and secretory activity, which, in turn, may predict treatment response [14]; after completion of the workup, patients should be staged based on the American Joint Committee on Cancer (AJCC) staging system 8^th^ edition [15].

**Figure 4:**
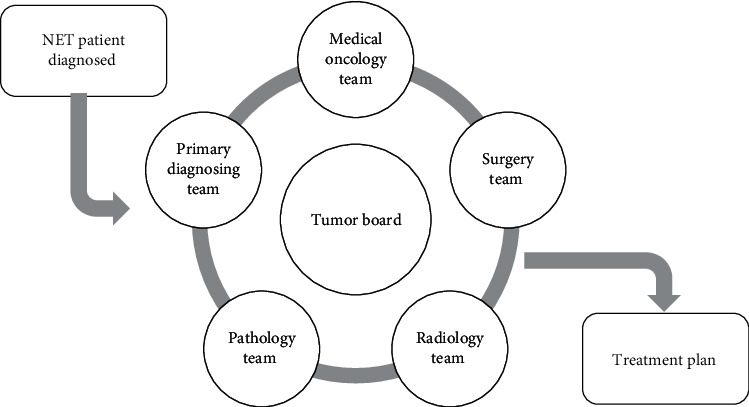
The NET tumor board and treatment plan construction.

At our hospital, every case with a histopathological diagnosis of NET should be presented within the weekly tumor board (in attendance of the primary diagnosing team, based usually on the tumor location, medical oncology, surgery team, radiology, and pathology) after completion of the workup—radiological and laboratory—and treatment plans are constructed for each case specifically, based on multiple parameters including the stage, performance status, and the clinical picture as shown in Figure 4.

**Figure 5:**
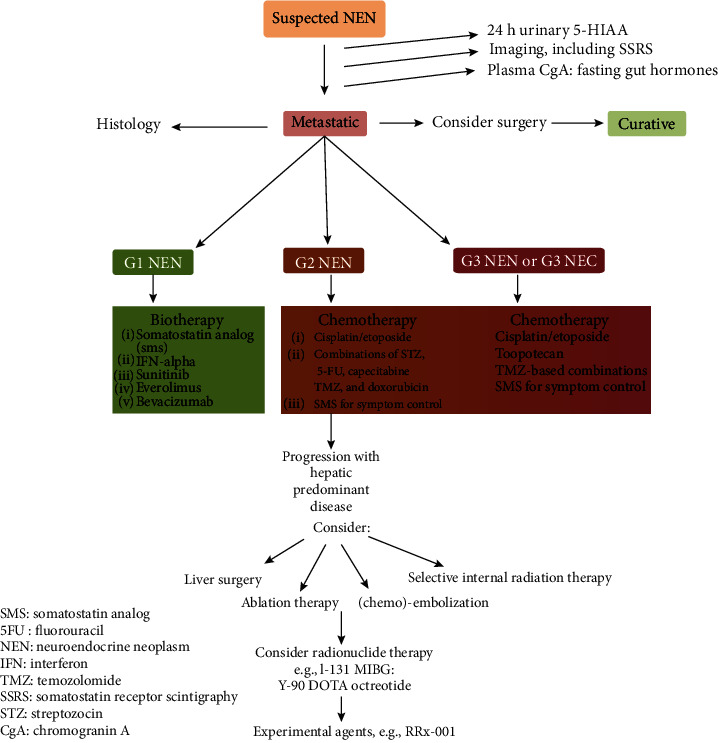
Different treatment modalities that can be used in the management of NETs [1].

There are different treatment modalities that can be used in the management of NET as shown in Figure 5 [1].

Actually, the optimal therapeutic sequence should be based on the evaluation of at least three major issues [16]:

- Tumor characterization (primary site, histological diagnosis, and staging)
- Patient's clinical status (performance status, clinical picture, prior treatments, and comorbidities)
- Defining the objectives of care

Fortunately, 5 of our patients were diagnosed in early stages and thus, these 5 patients were treated radically aiming for cure and they underwent radical surgeries; 2 cases underwent upfront right hemicolectomy, and 2 cases underwent appendectomy, whereas the last case underwent appendectomy, but based on the histopathological findings and the discussion of the case within the tumor board completion, right hemicolectomy was done. Based on the clinical picture and staging of these 5 cases, no adjuvant treatment was required. One case presented with a metastatic disease to the liver, and she presented in poor general condition, and unfortunately, she died without starting any treatment. All patients who did radical surgery were following in surgery and oncology clinics; follow-up consists of clinical examination and abdominal imaging (6 months after imaging and then as clinically indicated).

## 4. Conclusion

Despite the increasing number of patients diagnosed with GIT NET and also the increasing knowledge within this field, still, most of the cases are diagnosed in advanced stages; thus, a higher incidence of suspicion is required. Also, there are still some controversies regarding the sequence of systemic treatments used in the management of GIT NET that needs more clarification.

## Figures and Tables

**Table 1:** Summary of the data of the patients diagnosed with GIT NET at AFHSR.

Age, sex	Initial presentation	Site	Size	Diagnostic workup	Grade	Lymph node	Immunohistochemical study	Modality of treatment	Outcome
60, M	Acute intestinal obstruction	Ileocecal mass	45 × 20 mm	CT of the abdomen shows an enhancing mass at the ileocecal area including the appendix	Moderately differentiated arising from goblet cell carcinoid	Positive for metastasis	Synaptophysin +, chromogranin +, CK-20 +	Right Hemicolectomy	Alive and following with oncology
20, F	Appendicitis	Distal 1/3 of the appendix	4 × 3 mm	Follow-up CT was unremarkable	Grade 1, well differentiated	No	Synaptophysin +, chromogranin +, Ki-67 less than 3%	Appendectomy	Alive and following up with an oncologist
19, F	Appendicitis	Distal 1/3 of the appendix	1 × 2 mm	Follow-up CT was unremarkable	Grade 1, well differentiated	No	Synaptophysin +, chromogranin +, Ki-67 less than 2%	Appendectomy	Alive and following up with an oncologist
52, M	Acute intestinal obstruction	Ileocecal mass	15 mm	CT of the abdomen showed an enhancing mass at the ileocecal area including the appendix Follow-up CT of the abdomen showed a free anastomosis site	Grade1, well differentiated	No	Synaptophysin NA, chromogranin NA, Ki-67 less than 3%	Right hemicolectomy	Alive and following up with an oncologist
80, F	Abdominal pain and weight loss	Appendix	—	CT of the abdomen shows multiple liver lesions with an enlarged appendix Liver biopsy	Grade 1	Positive for metastasis	Synaptophysin +, chromogranin +, Ki-67 1-2%	—	Died
22, M	Appendicitis	Appendix	7 mm	Follow-up CT was unremarkable	Grade 1, well differentiated; tumor invades the muscle to periappendiceal fatty tissue, and there is perineural invasion	No	Synaptophysin +, chromogranin +, Ki-67 1%	Appendectomy followed by right hemicolectomy	Alive and following up with an oncologist
86, M	UGI bleeding	Stomach	—	Gastroscopy showed ugly-looking gastric ulcer	Grade 1	—	Synaptophysin NA, chromogranin NA, Ki-67 NA	—	Follow-up in another center
